# Challenges in Rehabilitation of a Tetanus Patient With Severe Complications

**DOI:** 10.7759/cureus.76494

**Published:** 2024-12-27

**Authors:** Masayoshi Seki, Mizuki Sugiyama, Takuya Maeda, Fumihito Kasai

**Affiliations:** 1 Department of Rehabilitation Center, Koto Toyosu Hospital, Showa University, Tokyo, JPN; 2 Department of Rehabilitation Medicine, School of Medicine, Showa University, Tokyo, JPN; 3 Department of Physical Therapy, School of Nursing and Rehabilitation Sciences, Showa University, Tokyo, JPN

**Keywords:** complications, intensive care, neurotoxin, rehabilitation, tetanus

## Abstract

Tetanus is a rare but life-threatening neurological disorder caused by neurotoxins produced by *Clostridium tetani*. Although mortality rates have significantly decreased with modern intensive care, severe cases remain challenging due to prolonged Intensive Care Unit (ICU) stays, complications, and rehabilitation barriers. We report the case of an 81-year-old male with a history of hypertension and femoral neck fracture who developed severe tetanus following a contaminated forehead laceration. Despite appropriate wound management, symptoms progressed rapidly, including trismus, generalized muscle spasms, opisthotonus, and respiratory failure requiring mechanical ventilation. Rehabilitation therapy began on the 10th day of hospitalization and focused on positioning techniques and passive range-of-motion exercises to reduce opisthotonus, preserve joint mobility, and prevent contractures. However, complications such as pneumonia, septic shock, and autonomic instability led to interruptions in therapy. By transfer on the 80th day, muscle tone had improved (Adductor Tone Rating: 2→1), and functional independence, measured by the Barthel Index, reached 10 points. Despite persistent spasticity in the upper limbs and finger contractures, early rehabilitation and multidisciplinary collaboration mitigated further functional decline. This case highlights the importance of initiating tailored rehabilitation early, even amidst life-saving interventions, to address the unique complexities of severe tetanus and prevent secondary complications such as disuse syndrome.

## Introduction

Pathophysiology

Tetanus is a neurological disorder characterized by generalized skeletal muscle spasms and rigidity caused by the exotoxin (tetanospasmin) produced by Clostridium tetani [1]. Tetanospasmin disrupts inhibitory neurotransmitter release (GABA and glycine) at the spinal cord and brainstem, leading to uncontrolled motor neuron excitation, which causes persistent muscle spasms and rigidity. Tetanus bacillus enters the body through wounds, germinates, and proliferates under anaerobic conditions at the wound site, producing toxins. If the wound site is close to the central nervous system, the incubation period is shorter, and symptoms tend to be more severe, with a higher risk of complications and mortality [2-3]. Despite the introduction of the tetanus vaccine, approximately 100 individuals in Japan still contract tetanus annually, resulting in 5-9 deaths [4]. Globally, over 50,000 new cases and 20,000 deaths occur annually, primarily in low-resource settings, due to limited vaccination coverage and medical access [5].

Clinical symptoms

Tetanus typically presents with muscle spasms and stiffness. Initially, muscles innervated by cranial nerves are affected, leading to difficulty in mouth opening (trismus), spasmic smiles (risus sardonicus), laryngeal spasms, and swallowing difficulties. Over the next 1-2 days, stiffness spreads progressively to the arms, trunk, and eventually, the entire body, resulting in generalized opisthotonus, a hallmark posture characterized by severe hyperextension of the back, neck, and limbs. Even minor sensory stimuli can trigger prolonged and painful spasms. Overactivity of the sympathetic nervous system leads to autonomic instability, manifesting as severe tachycardia (heart rate >120 bpm), blood pressure fluctuations (e.g., 160/90 to 90/50 mmHg), and diaphoresis.

Complications

Severe complications include respiratory failure, autonomic dysregulation, pneumonia, rhabdomyolysis, deep vein thrombosis (DVT), and pressure ulcers, all of which contribute to the high mortality rate [6]. These complications hinder rehabilitation and exacerbate the already challenging recovery process. Early rehabilitation therapy for patients with severe tetanus has been shown to lead to favorable outcomes by reducing complications and improving functional independence [7].

Rationale for this report

Despite established protocols for wound care, antitoxin administration, and supportive therapy, tetanus cases requiring prolonged intensive care unit (ICU) care pose unique challenges. This case highlights the critical role of multidisciplinary collaboration and tailored rehabilitation in managing opisthotonus and autonomic instability.

This article was previously presented as a meeting abstract at the 5th Annual Autumn Meeting of the Japanese Association of Rehabilitation Medicine held from November 12 to 14, 2021.

## Case presentation

Case

81-year-old male Past medical history: Hypertension and left femoral neck fracture (hip replacement surgery) Present illness: The patient fell on the roadside near his home, hit his forehead on the curb, and was transported to the emergency department. A laceration was observed on the forehead with noticeable contamination, leading to wound cleansing and suturing. Tetanus toxoid was administered, and the patient was discharged. A plan for observation and oral antibiotics was prepared. However, the next day, he was brought to the emergency department complaining of difficulty walking and limb weakness and was admitted with a provisional diagnosis of tetanus. On the second day of hospitalization, debridement was performed due to persistent contamination at the wound site. Difficulty in swallowing and mouth opening appeared on the third day, and on the seventh day, respiratory failure occurred, leading to endotracheal intubation. Rehabilitation therapy was initiated on the tenth day of hospitalization.

Rehabilitation progress

**Figure 1:**
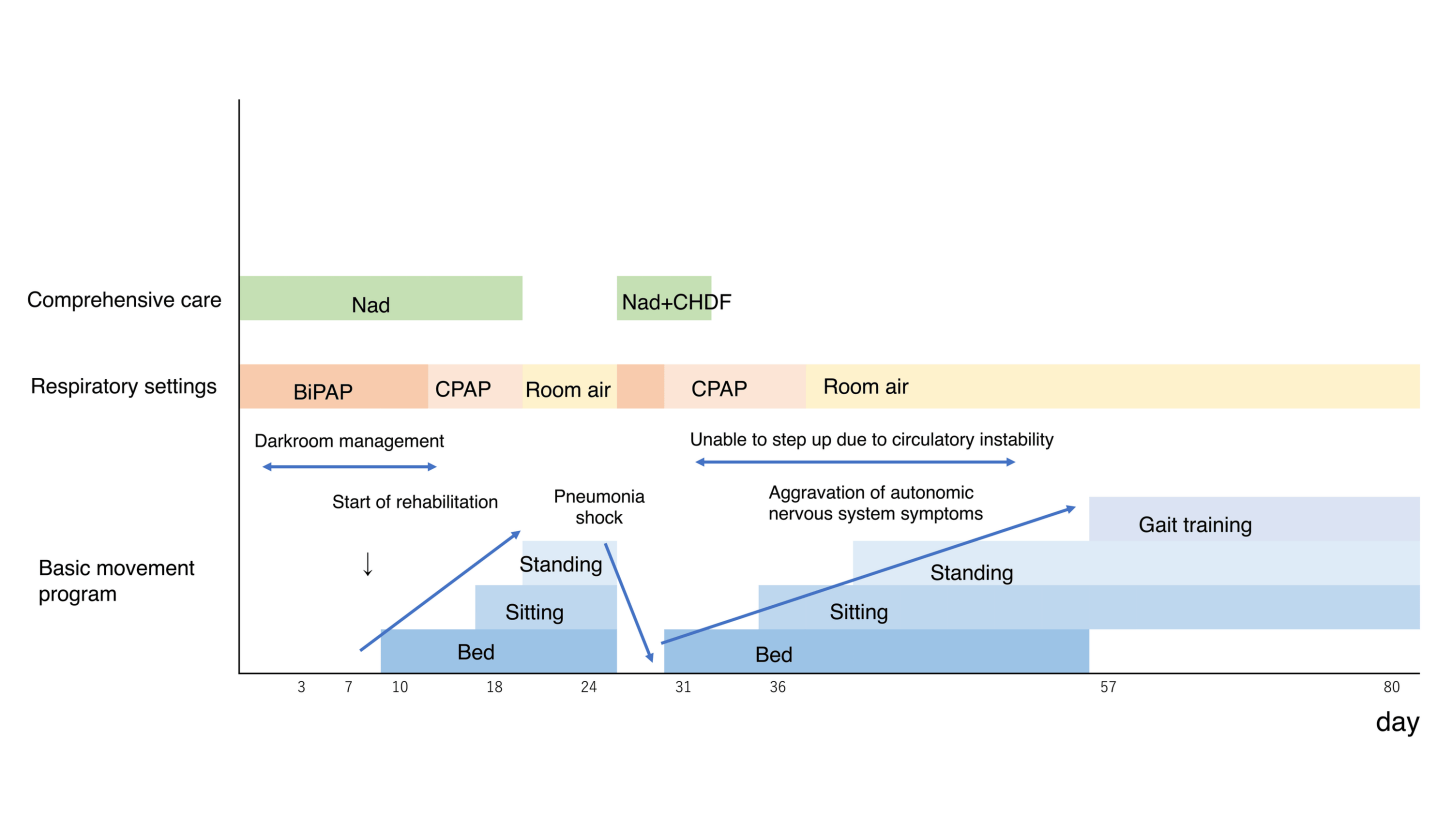
Overall management and rehabilitation progress CHDF: Continuous Hemodiafiltration; Nad: Noradrenaline; CPAP: Continuous Positive Airway Pressure; BiPAP: Bilevel Positive Airway Pressure.

The course after admission is shown in Figure 1.

Initial assessment and early intervention

Upon initial consultation, the patient was evaluated under dim light conditions to minimize sensory stimuli that could exacerbate muscle spasms or autonomic instability.

Clinical Observations

Consciousness: Glasgow Coma Scale (GCS): E1VtM1. Muscle Tone Measurements: Modified Ashworth Scale (MAS): 1 for triceps brachii. Adductor Tone Rating (ATR): 2 for hip adductors

**Figure 2:**
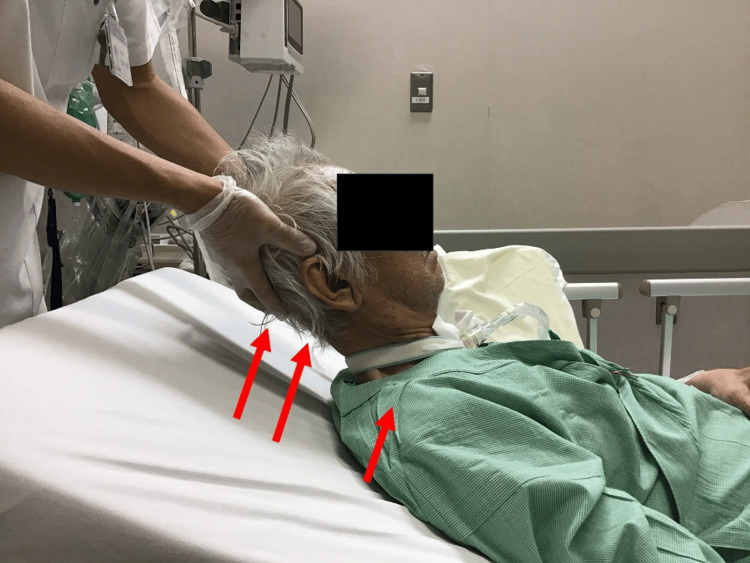
Increased neck muscle stiffness The figure illustrates increased neck muscle stiffness and muscle tone in the extensor muscles of the neck and trunk. This increase caused the neck and trunk to lift as a single unit when the posterior neck was supported.

Increased muscle tension was observed in the posterior neck and back. Extension of the neck led to simultaneous elevation of the trunk when the patient attempted to lift his head, indicating strong extensor tone in the neck and trunk. Immediate intervention was required to prevent further complications (Figure 2).

Intervention (day 10-18)

Positioning Strategies

Semi-recumbent positioning with supportive cushions was implemented to reduce opisthotonus and maintain limb alignment. Particular attention was given to minimizing neck extensor tension using head-neck support cushions.

Passive Range of Motion (PROM) Exercises

Gentle PROM exercises targeting hip and knee flexion-extension were performed twice daily (15 repetitions per session) to prevent joint contractures and maintain muscle length.

Preventive Measures

Regular two-hour repositioning to prevent pressure ulcers. Elastic compression stockings and a pressure-relieving mattress were employed to mitigate the risk of deep vein thrombosis (DVT) and skin breakdown.

Progressive Interventions and Challenges

Day 15: Consciousness improved to GCS: E1VtM6, allowing for increased rehabilitation intensity. Difficulty in eye-opening persisted, necessitating the gradual reintroduction of sensory stimuli.

Intervention (day 18-24)

Seated Training

Seated training was initiated with auditory stimuli to improve posture awareness and engagement. Trunk rotation movements and frequent position changes were implemented to relax back extensors. However, extreme increases in extensor muscle tension were observed during antigravity activities.

Ambulation Training

Ambulation training was cautiously attempted but was limited by prominent extensor spasms in the back.

Adaptation During Septic Shock (Day 24-30)

Active training was paused due to hemodynamic instability caused by septic shock. Alternative interventions included: 1) Gentle breathing exercises to maintain respiratory function. 2) Bed-based PROM exercises to prevent joint stiffness and muscle atrophy.

Resumption of Rehabilitation and Outcomes

Rehabilitation resumed on day 31, focusing on maintaining functional gains while addressing residual autonomic instability (e.g., tachycardia, blood pressure fluctuations) and respiratory challenges.

Intervention (day 31-80)

Range of Motion (ROM) Exercises and Targeted Stretching

PROM exercises were intensified to include trunk rotation and back extensor elongation.

Standing and Basic Walking Exercises

Standing exercises were reintroduced with close hemodynamic monitoring. Basic walking exercises were performed despite persistent upper limb spasticity and finger flexion contractures.

Multidisciplinary Collaboration

Nurses assisted in maintaining consistent positioning and PROM exercises outside scheduled rehabilitation sessions, ensuring continuity of care.

Outcome at transfer (day 80)

Muscle Tone

MAS:1 (Triceps brachii; stable), ATR: Improved from 2 to 1. Functional Independence: Barthel Index (BI): Improved to 10 points, reflecting slight but measurable functional recovery. Consciousness: GCS: Maintained at E1VtM6.

## Discussion

In countries with access to intensive care, mortality rates for tetanus have decreased significantly from 44% to 15% with acute-phase treatments, including mechanical ventilation and antitoxin administration [8]. However, in resource-limited settings, where prolonged intensive care and ventilation facilities are unavailable, mortality rates for severe tetanus remain high, exceeding 50%, with airway obstruction, respiratory failure, and renal failure as leading causes of death. Mortality rates also increase with age, particularly surpassing 50% in individuals over 60 years of age [3]. In this case, the patient was 81 years old, placing him at particularly high risk for severe outcomes, further complicated by a short incubation period (three days), indicative of a high toxin burden.

Tetanus presents with various clinical symptoms, ranging from mild trismus and localized rigidity to generalized spasms, opisthotonus, and autonomic instability (Table 1) [9].

**Table 1:** Clinical classifications of tetanus

Classification	Description
Generalized	Begins with trismus and risus sardonicus (spasm of the facial muscles), then proceeds to generalized spasms and opisthotonos
Localized	Muscle rigidity limited to the site of spore inoculation
Cephalic	Form of localized tetanus affecting cranial nerves, often following a head injury
Neonatal	Generalized tetanus in newborns resulting from infection of the umbilical stump

According to the Ablett classification (Table 2) [9], this case was classified as Grade IV tetanus, characterized by severe generalized spasms and violent autonomic disturbances, including significant blood pressure fluctuations and sustained tachycardia. These features aligned with the patient’s presentation of opisthotonus, severe extensor tone, and autonomic instability, all of which posed unique challenges to rehabilitation.

**Table 2:** Ablett classification of severity of tetanus

Grade	Clinical features
Ⅰ	Mild: mild to moderate trismus; general spasticity; no respiratory embarrassment [respiratory distress]; no spasms; little or no dysphagia
Ⅱ	Moderate: moderate trismus; well-marked rigidity; mild to moderate but short spasms; moderate respiratory embarrassment with an increased respiratory rate greater than 30 [breaths/min]; mild dysphagia
Ⅲ	Severe: severe trismus; generalized spasticity; reflex prolonged spasms; increased respiratory rate greater than 40 [breaths/min]; apneic spells; severe dysphagia; tachycardia greater than 120 [beats/min]
Ⅳ	Very severe: grade III and violent autonomic disturbances involving the cardiovascular system; severe hypertension and tachycardia alternating with relative hypotension and bradycardia, either of which may be persistent

Challenges in rehabilitation for severe tetanus

Rehabilitation for severe tetanus is often delayed due to the critical need for life-saving management, resulting in prolonged immobilization, increased extensor muscle tone, and a heightened risk of disuse syndrome. In this case, we implemented early rehabilitation strategies despite the patient’s critical condition, emphasizing positioning techniques and PROM exercises initiated within the first 24-48 hours of stabilization.

Positioning aimed to maintain joint alignment, minimize opisthotonus, and prevent further flexion contractures. Specific adjustments, such as semi-recumbent positioning and the use of supportive cushions, played a crucial role in mitigating extensor activity in the neck and back. PROM exercises, focusing on gentle hip and knee flexion-extension movements (15 repetitions/session, twice daily), were prioritized to preserve joint mobility. As a result, the patient progressed to standing exercises by Day 22 of hospitalization, an outcome that underscores the importance of early mobilization even in severe cases.

Impact of complications on rehabilitation

Additionally, severe tetanus is associated with significant autonomic instability [10-11], with the sympathetic nervous system being the most affected. Clinically, increased sympathetic tone can cause sustained tachycardia and hypertension, which present substantial challenges during acute-phase management. These autonomic disturbances often prolong the use of vasopressors and complicate the timing and intensity of rehabilitation interventions. In this case, autonomic instability was characterized by recurrent blood pressure fluctuations and persistent tachycardia, necessitating careful monitoring and limiting the progression of rehabilitation therapy during the early phases.

The transition from life-saving management to active rehabilitation therapy was particularly challenging, as systemic disuse syndrome and ROM limitations became pronounced due to prolonged immobilization. Despite these challenges, early initiation of PROM exercises and positioning strategies minimized further deterioration. However, persistent autonomic instability underscored the need for individualized and adaptive rehabilitation protocols tailored to the patient’s fluctuating hemodynamic status.

Despite favorable initial progress, complications such as pneumonia and septic shock prolonged bed rest and limited active rehabilitation efforts. During this period, alternative interventions, including gentle breathing exercises and bed-based ROM techniques, were implemented to maintain muscle length and circulation, preventing further deterioration. This adaptive strategy highlights the importance of flexibility in rehabilitation protocols, particularly in ICU settings where patient stability can fluctuate.

Multidisciplinary collaboration

A multidisciplinary approach was critical to achieving progress in this case. Nurses played a pivotal role in maintaining positioning protocols outside scheduled rehabilitation sessions, ensuring consistent patient care, and minimizing complications like pressure ulcers and DVT. ICU staff provided continuous monitoring of autonomic stability, creating a safe environment for implementing targeted interventions. This collaboration highlights the essential role of interdisciplinary communication in optimizing rehabilitation outcomes for severe tetanus patients.

Comparison with literature and outcomes

Previous studies have emphasized the importance of early mobilization and structured positioning protocols to mitigate the effects of disuse syndrome in ICU patients with severe tetanus [12-13]. In this case, PROM exercises and positioning effectively stabilized extensor tone, as evidenced by improvements in the ATR from 2 to 1 and the prevention of further contracture progression. Additionally, functional independence, measured using the Barthel Index, increased to 10 points by the time of transfer, reflecting measurable progress despite the severity of the condition.

Compared to typical severe tetanus cases, where rehabilitation is often delayed due to autonomic instability and prolonged ICU stays, this case demonstrates the potential benefits of early intervention. While persistent upper limb spasticity and finger contractures remained challenges, targeted interventions successfully minimized further deterioration and supported functional recovery.

Clinical implications

This case underscores the importance of early rehabilitation, even amidst life-saving interventions, to reduce the risks of disuse syndrome and joint contractures in severe tetanus patients. Future cases could benefit from structured rehabilitation protocols tailored to individual clinical conditions, emphasizing early PROM exercises, effective positioning strategies, and interdisciplinary collaboration.

This case highlights the unique challenges of rehabilitation in severe tetanus characterized by opisthotonus, autonomic instability, and prolonged ICU care. Early and structured rehabilitation interventions, including PROM exercises and targeted positioning strategies, played a crucial role in stabilizing muscle tone and minimizing the risk of contractures, despite the patient’s critical condition. Measured improvements in muscle tone (ATR: 2→1) and functional independence (Barthel Index: 10 points) demonstrate the benefits of initiating PROM exercises and structured positioning protocols early in the acute phase.

The successful progression of this case emphasizes that early initiation of PROM exercises and structured positioning protocols should be prioritized, even in critically ill patients, to prevent secondary complications such as joint contractures and disuse syndrome. Additionally, close interdisciplinary collaboration, including ICU staff ensuring autonomic stability, nurses maintaining consistent positioning, and rehabilitation specialists tailoring interventions, was essential in maintaining rehabilitation continuity and optimizing patient outcomes.

This report underscores the importance of integrating rehabilitation efforts early during the acute management of severe tetanus. By addressing complications promptly and fostering coordinated care, rehabilitation outcomes can be improved, even in complex cases like this one.

## Conclusions

This case highlights the critical importance of early and structured rehabilitation in managing severe tetanus, characterized by opisthotonus, autonomic instability, and prolonged ICU stays. Despite the patient’s advanced age, short incubation period, and associated high risk of complications, early interventions, including PROM exercises, targeted positioning strategies, and multidisciplinary collaboration, successfully minimized secondary complications such as joint contractures and disuse syndrome.

Improvements in muscle tone and functional independence, as evidenced by the reduction in the ATR and increased Barthel Index, underscore the efficacy of initiating rehabilitation efforts even in critically ill patients. Furthermore, this case emphasizes the value of individualized and adaptive rehabilitation protocols that accommodate the fluctuating clinical status of severe tetanus patients.

The findings advocate for the integration of early rehabilitation into acute-phase management of severe tetanus, supported by close interdisciplinary communication and tailored interventions. By adopting such approaches, healthcare teams can optimize outcomes and enhance recovery trajectories for patients facing this challenging condition.
